# Genome-wide association and genomic prediction identifies associated loci and predicts the sensitivity of Tobacco ringspot virus in soybean plant introductions

**DOI:** 10.1186/s12864-016-2487-7

**Published:** 2016-02-29

**Authors:** Hao-Xun Chang, Patrick J. Brown, Alexander E. Lipka, Leslie L. Domier, Glen L. Hartman

**Affiliations:** Department of Crop Sciences, University of Illinois, Urbana, IL 61801 USA; USDA–Agricultural Research Service, Urbana, IL 61801 USA; National Soybean Research Center, University of Illinois, 1101 W. Peabody Dr., Urbana, IL 61801 USA

**Keywords:** Genome association and prediction integrated tool (GAPIT), Genome-wide association studies (GWAS), Genomic prediction, Single nucleotide polymorphism (SNP), Soybean (*Glycine max*), *Tobacco ringspot virus* (TRSV)

## Abstract

**Background:**

Genome-wide association study (GWAS) is a useful tool for detecting and characterizing traits of interest including those associated with disease resistance in soybean. The availability of 50,000 single nucleotide polymorphism (SNP) markers (SoySNP50K iSelect BeadChip; www.soybase.org) on 19,652 soybean and wild soybean plant introductions (PIs) in the USDA Soybean Germplasm Collection allows for fast and robust identification of loci associated with a desired phenotype. By using a genome-wide marker set to predict phenotypic values, genomic prediction for phenotype-unknown but genotype-determined PIs has become possible. The goal of this study was to describe the genetic architecture associated with sensitivity to *Tobacco ringspot virus* (TRSV) infection in the USDA Soybean Germplasm Collection.

**Results:**

TRSV-induced disease sensitivities of the 697 soybean PIs were rated on a one to five scale with plants rated as one exhibiting mild symptoms and plants rated as five displaying terminal bud necrosis (i.e., bud blight). The GWAS identified a single locus on soybean chromosome 2 strongly associated with TRSV sensitivity. Cross-validation showed a correlation of 0.55 (*P* < 0.01) to TRSV sensitivity without including the most significant SNP marker from the GWAS as a covariate, which was a better estimation compared to the mean separation by using significant SNPs. The genomic estimated breeding values for the remaining 18,955 unscreened soybean PIs in the USDA Soybean Germplasm Collection were obtained using the GAPIT R package. To evaluate the prediction accuracy, an additional 55 soybean accessions were evaluated for sensitivity to TRSV, which resulted in a correlation of 0.67 (*P* < 0.01) between actual and predicted severities.

**Conclusion:**

A single locus responsible for TRSV sensitivity in soybean was identified on chromosome 2. Two leucine-rich repeat receptor-like kinase genes were located near the locus and may control sensitivity of soybean to TRSV infection. Furthermore, a comprehensive genomic prediction for TRSV sensitivity for all accessions in the USDA Soybean Germplasm Collection was completed.

**Electronic supplementary material:**

The online version of this article (doi:10.1186/s12864-016-2487-7) contains supplementary material, which is available to authorized users.

## Background

*Tobacco ringspot virus* (TRSV), a single-stranded bipartite RNA virus, is one of the most destructive viral pathogens of soybean (*Glycine max* (L.) Merr.) [1]. Soybean plants infected with TRSV are generally stunted, leaflets may be dwarfed and rolled, buds may become brown, necrotic and brittle, and terminal buds may form a crook and die. Plants infected at early developmental stages often produce undeveloped flowers that impact fertilization resulting in aborted pods and yield losses ranging from 25 to 100 % [1, 2]. TRSV is transmitted through infected seeds or by vectors, including the dagger nematode (*Xiphinema americanum* Cobb), grasshoppers (*Melanoplus differentialis* Thomas), thrips (*Thrips tabaci* Lindeman) and tobacco flea beetles (*Epitrix hirtipennis* Melsheimer) [1]. Transmission by seed and nematodes may be the most common pathways of infection [3]. TRSV has a broad host range that includes many plant genera, and has been reported on soybean in most soybean producing states in the USA and in Australia, Canada, the People’s Republic of China, and Russia [1].

There are few options for managing TRSV outbreaks. Resistance to TRSV has not been described for commercial soybean cultivars, and may not be available based on the absence of TRSV resistance in a set of 52 lines that are the ancestors to most North American public cultivars [4]. In another study, TRSV resistance was reported in wild soybean (*Glycine soja* Siebold & Zucc.) as three out of 630 plant introductions (PIs) displayed only mild symptoms when infected by TRSV [5]. No further studies have been conducted to determine the inheritance of the potential sources of resistance in *G. soja*. However, one study crossed the soybean cultivar Young and PI416937 and identified a major quantitative trait locus (QTL) that explained 82 % of the phenotypic variation of TRSV resistance. This QTL was located on chromosome 13 between 25 Mb to 28 Mb between markers K644_1 and Satt510 based on Williams82 assembly version 1 (Gmax1.01) [6]. In addition, when *Arabidopsis thaliana* (L.) Heynh. was inoculated with TRSV, most ecotypes were tolerant to TRSV, but some ecotypes, such as Estland, displayed lethal systemic necrosis [7], which resembled bud blight of soybean. An allele of *TTR1* (Tolerance to *Tobacco ringspot virus* 1), which encodes a protein with Toll/interleukin-1 receptor, nucleotide-binding site and leucine-rich repeat (TIR-NB-LRR) domains was reported to control tolerance to TRSV in *A. thaliana* [8]. Comparison of protein sequence alignments between tolerant and sensitive *A. thaliana* ecotypes identified different amino acid residues in the LRR region between sensitive *TTR1* and tolerant *ttr1* alleles. When the TRSV-tolerant *A. thaliana* ecotype Col-0 was transformed with the *TTR1* allele from the sensitive Estland ecotype, the resulting plants were sensitive to TRSV infection. In contrast, when Col-0 was transformed with *TTR1* that contained single amino acid substitutions at different locations, only L956S and K1124Q escaped the necrosis symptoms, suggesting that the leucine (L956) and lysine (K1124) residues in the *TTR1* gene were needed for TRSV sensitivity and for displaying lethal systemic necrosis [8].

The genome-wide association study (GWAS) is a statistical analysis that associates variation across the entire genome with phenotypes [9, 10]. In the case of soybean, GWASs have been used to identify loci associated with agronomic traits [11], abiotic stress [12], and disease resistance including Phytophthora root rot (*Phytophthora sojae* Kaufman & Gerdman) [13], Sclerotinia stem rot (*Sclerotinia sclerotiorum* (Lib.) de Bary) [14–16], soybean cyst nematode (*Heterodera glycines* Ichinohe) [17–19], and sudden death syndrome (*Fusarium virguliforme* Akoi, O’Donnell, Homma &. Lattanzi) [20]. With the availability of SoySNP50K single nucleotide polymorphism (SNP) markers [21], genomic information for over 19,000 accessions in the USDA Soybean Germplasm Collection can be utilized to identify genes underlying many traits, including resistance or tolerance to TRSV.

The goal of this study was to identify regions of the soybean genome associated with sensitivity to TRSV infection based on a subset of the PIs in the USDA Soybean Germplasm Collection. For these analyses, 697 soybean PIs were phenotyped TRSV sensitivity and GWAS was performed, which identified a single locus associated with sensitivity to TRSV infection. Moreover, we applied genomic prediction to estimate TRSV sensitivities for the unscreened 18,955 soybean PIs in the USDA Soybean Germplasm Collection, and evaluated the accuracy of genomic prediction. To our knowledge, this work constitutes the first GWAS and genomic prediction study for TRSV sensitivity in soybean.

## Results

### Sensitivity of soybean PIs to TRSV infection

**Fig. 1:**
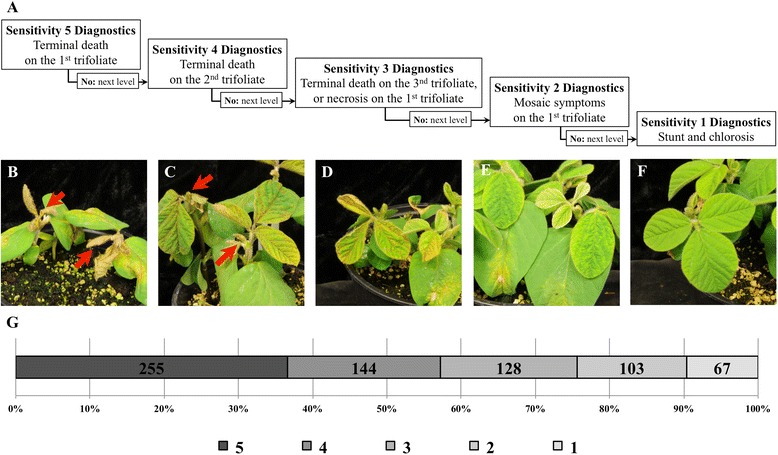
Sensitivity of soybean accessions to TRSV infection. **a** Decision tree for determining TRSV sensitivities one to five. **b** Red arrows indicate necrotic reactions on different trifoliates. When the first trifoliate became necrotic, plants were rated with a sensitivity of five. **c** Necrosis of the second trifoliate at the bud stage typifies sensitivity four. **d** Plants that had necrotic spots on first trifoliate leaves belonged to sensitivity three. **e** Plants in sensitivity two displayed clear mosaic symptoms on the first trifoliate. **f** Plants in sensitivity one were stunted with chlorosis. **g** The distribution of TRSV sensitivity on 697 soybean accessions

All 697 soybean PIs evaluated were susceptible to TRSV and showed symptoms. At ten days post inoculation, most soybean plants infected with TRSV were stunted and displayed a range of foliar symptoms (Fig. 1a). Plants were separated into sensitivity categories of one to five based on the severity of their symptoms. Plants that displayed terminal necrosis on the first or second trifoliates were classified as a sensitivity of five or four, respectively (Fig. 1b, c). Plants classified as sensitivity three had necrotic spots in the first or second trifoliate that generally started at the leaf margin or had terminal necrosis of third trifoliates (Fig. 1d). Plants with mosaic foliar symptoms on first trifoliates were categorized as sensitivity two (Fig. 1e) Plants with sensitivities of one were slightly stunted with mild chlorosis (Fig. 1f). More than 50 % of the PIs were classified as sensitivity four or five, and only 67 PIs were classified as sensitivity one (Fig. 1g).

### GWAS to identify QTL associated with sensitivity to TRSV

**Fig. 2:**
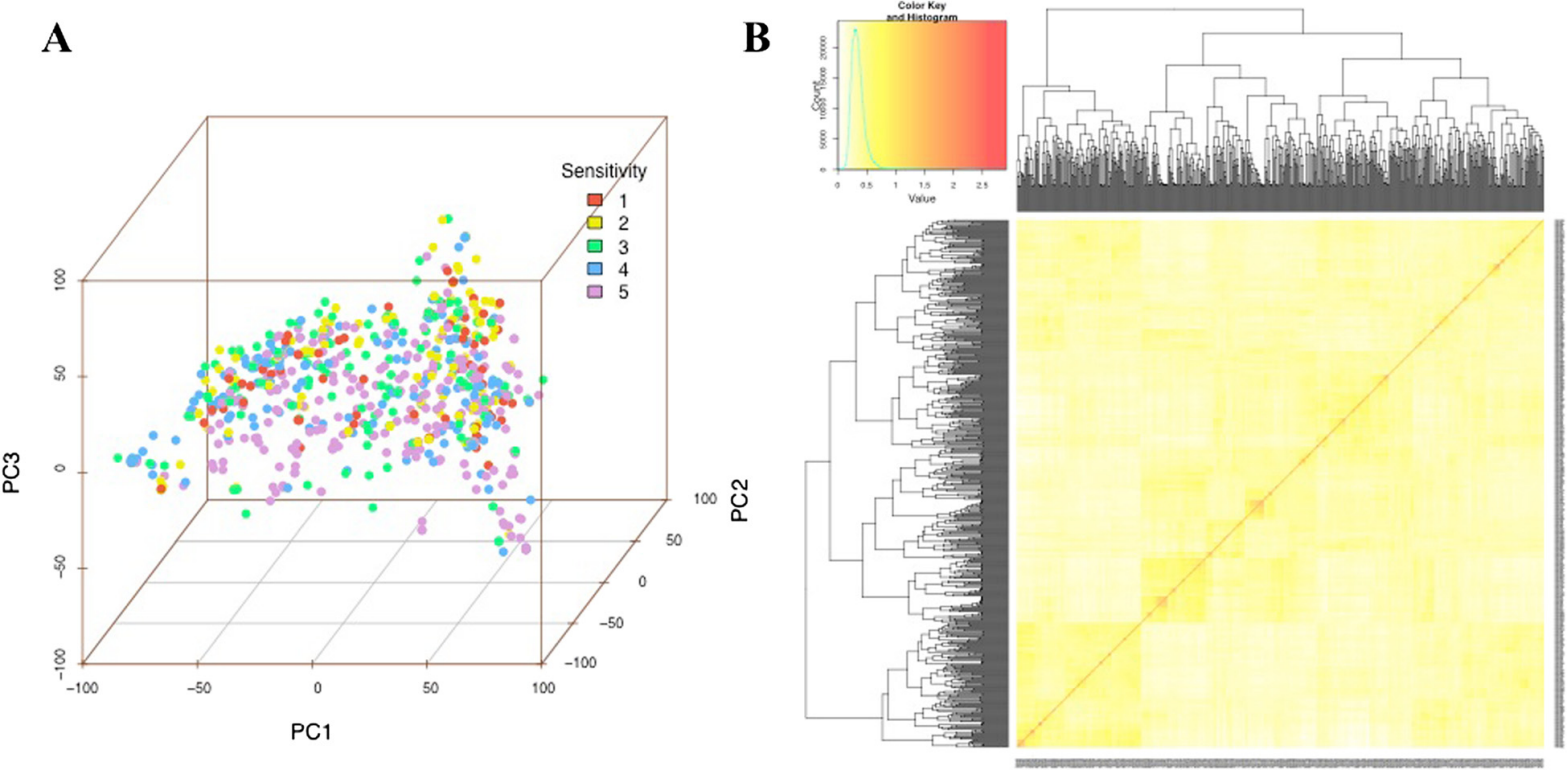
Principal component and kinship analyses of soybean genetic data. **a** The first three principal components of the 30,697 SNPs used in the genome-wide association study (GWAS) indicates little population structure among the 697 tested accessions. The different colors of dots indicate differing TRSV sensitivity values. **b** A heatmap of the kinship matrix of the 697 soybean accessions calculated from the same 30,697 SNPs used in the GWAS suggests low levels of relatedness among the 697 individuals

**Fig. 3:**
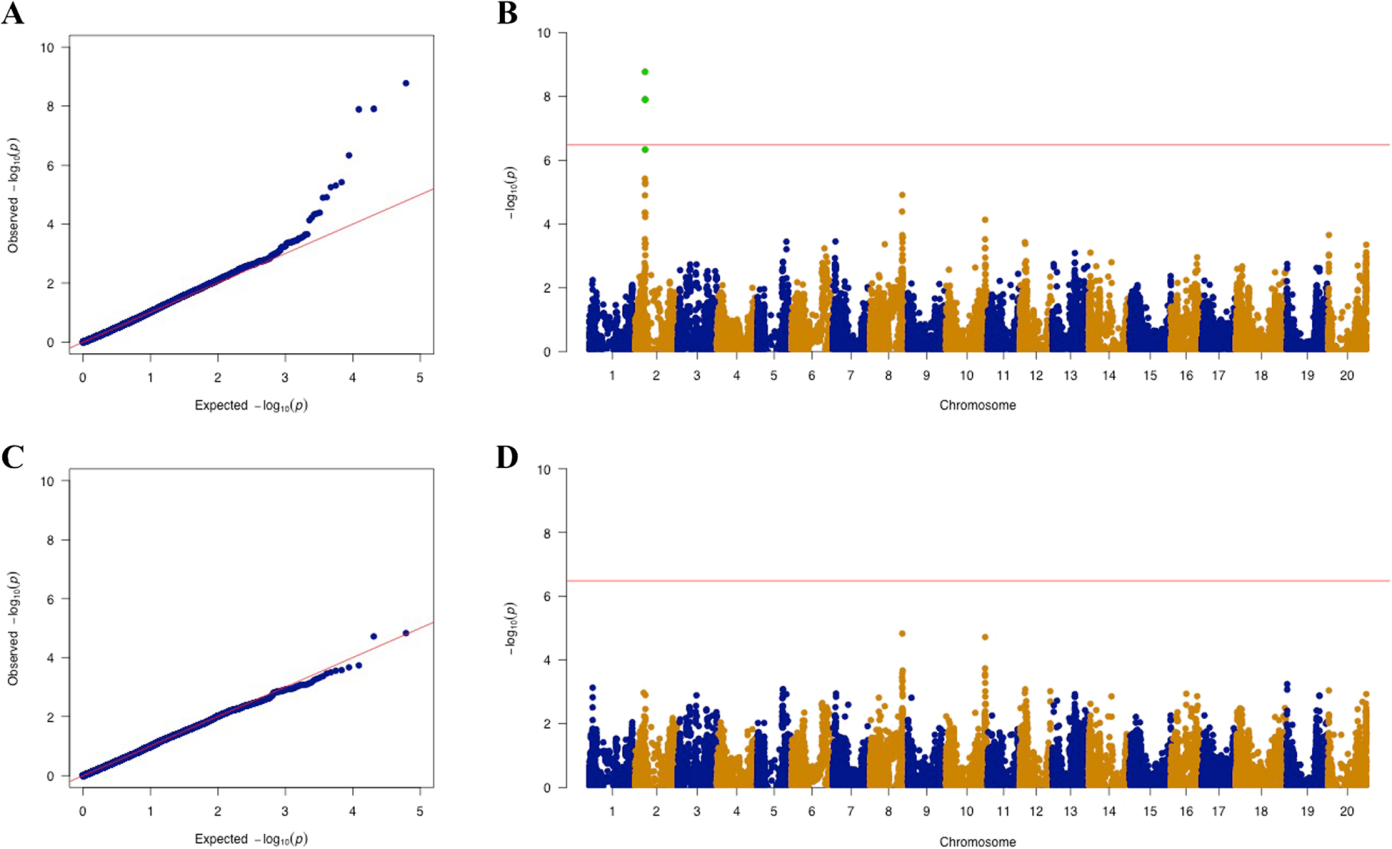
QQ-plots and Manhattan plots for TRSV sensitivities. **a** QQ-plot from the initial GWAS scan. Most *P*-values were similar to the expected diagonal in the QQ-plot, which indicates the appropriateness of the GWAS model. **b** A single QTL that exceeded genome-wide significance was identified on chromosome 2. Red line indicates Bonferroni-corrected threshold with an experimental type I error rate at 0.05. Significant SNPs at a false discovery rate of 1 % are highlighted in green. **c** QQ-plot from a second GWAS scan using a model that included the peak SNP from the initial GWAS scan (ss715581043) as a covariate. **d** The Manhattan plot with one covariate in the GWAS model identified no additional significant genomic signals

The Bayesian information criterion (BIC)-based model selection procedure [22] indicated that no principal components (PCs) were required to control for population structure in the GWAS model (Table 1). This result underscored our findings that the principal component analysis did not detect distinct subpopulations among the selected 697 soybean PIs (Fig. 2a). Therefore, no PC was included, but a heatmap of the kinship matrix with genetic relatedness among the 697 soybean PIs (Fig. 2b) was included in the mixed linear model for GWAS. Since the observed and expected *P*-values differed substantially only for a few SNPs, the quantile-quantile (QQ) plot supported the appropriateness of the GWAS model (Fig. 3a). The GWAS identified a single locus associated with TRSV sensitivity that exceeded the Bonferroni-corrected *α* = 0.05 threshold on chromosome 2 (Fig. 3b). Four SNPs in this region were significant at a 1 % false discovery rate (FDR). Individually, these SNPs accounted for 3 to 4 % of the variance in the GWAS model. Although most of the significant SNPs were located within a genomic region on chromosome 2, one SNP on chromosome 8 was significantly associated with TRSV sensitivity at 5 % FDR (Table 2). To confirm and detect other potential minor signals, the GWAS was reran with the most significant SNP on chromosome 2 (ss715581043) included as a covariate. The resulting QQ-plot showed that the observed *P*-values followed the expected *P*-values (Fig. 3c), and no additional SNPs identified at 5 % FDR (Fig. 3d). The result indicated that fixation of ss715581043 as a covariate explained most of the genetic contribution to the overall phenotypic variation and suggested only one locus on chromosome 2 was responsible for TRSV sensitivity in soybean. The four SNPs displaying peak associations with TRSV sensitivity in the initial GWAS defined a genomic region of approximately 130 kb between 12,089,749 bp to 12,219,313 bp on chromosome 2 (Table 2). Within this region, there were two candidate genes (Glyma02g13460 and Glyma02g13470) that may be involved in plant defense responses. Both encode leucine-rich repeat receptor-like kinases (LRR-RLKs) (Table 3).

**Table 1:** Bayesian information criterion (BIC)-based model selection. The model with the largest BIC value is optimal

Principal components	BIC	log(Likelihood Function)
0	−1125.50	−1115.68
1	−1128.76	−1115.67
2	−1131.94	−1115.58
3	−1133.62	−1113.98
4	−1136.89	−1113.98
5	−1140.16	−1113.97

**Table 2:** Top-ten SNPs in the genome-wide association study without any peak SNP covariates

SNP (ss_id)	Chromosome	Position	MAF	*P*-value	R^2^ of Model without SNP	R^2^ of model with SNP	FDR-adjusted *P*-value
ss715581043	2	12,089,749	0.416	1.68 × 10^−9^	0.22	0.26	5.16 × 10^−5^
ss715581049	2	12,190,975	0.415	1.23 × 10^−8^	0.22	0.26	1.31 × 10^−4^
ss715581051	2	12,206,518	0.422	1.28 × 10^−8^	0.22	0.26	1.31 × 10^−4^
ss715581052	2	12,219,313	0.480	4.60 × 10^−7^	0.22	0.25	3.53 × 10^−3^
ss715581033	2	11,974,580	0.493	3.76 × 10^−6^	0.22	0.24	2.31 × 10^−2^
ss715581062	2	12,327,212	0.453	4.90 × 10^−6^	0.22	0.24	2.43 × 10^−2^
ss715581054	2	12,235,906	0.481	5.54 × 10^−6^	0.22	0.24	2.43 × 10^−2^
ss715601789	8	40,952,506	0.473	1.22 × 10^−5^	0.22	0.24	4.30 × 10^−2^
ss715581036	2	12,036,555	0.430	1.26 × 10^−5^	0.22	0.24	4.30 × 10^−2^
ss715601747	8	40,684,679	0.433	4.07 × 10^−5^	0.22	0.24	1.19 × 10^−1^

**Table 3:** Genes between 12,089,749 bp to 12,206,518 bp on soybean chromosome 2^a^

Gene ID	Position (start..end)	Annotation
Glyma.02 g121600	12,084,714..12,089,043	K-box region and MADS box transcription factor
Glyma.02 g121700	12,093,068..12,095,722	RING/U-box Zinc finger, C3HC4 typeprotein
Glyma.02 g121800	12,106,073..12,107,969	Adenine nucleotide alpha hydrolases-like superfamily protein
Glyma.02 g121900	12,112,034..12,115,054	Leucine-rich repeat (Malectin-like) protein kinase family protein
Glyma.02 g122000	12,115,287..12,118,397	Leucine-rich repeat (Malectin-like) protein kinase family protein
Glyma.02 g122100	12,134,374..12,137,612	Heavy metal transport/detoxification superfamily protein
Glyma.02 g122200	12,141,974..12,149,160	Chaperone DnaJ-domain superfamily protein
Glyma.02 g122300	12,143,960..12,145,950	Putative unknown protein
Glyma.02 g122400	12,150,906..12,151,220	Putative unknown protein
Glyma.02 g122500	12,158,735..12,163,084	ACT domain repeat 4
Glyma.02 g122600	12,195,454..12,196,275	FRS (FAR1 Related Sequences) transcription factor family

^a^Gene ID, position, and annotation were based on soybean genome assembly version Glyma.Wm82.a2 (Glyma2.0)

### Genomic prediction

**Fig. 4:**
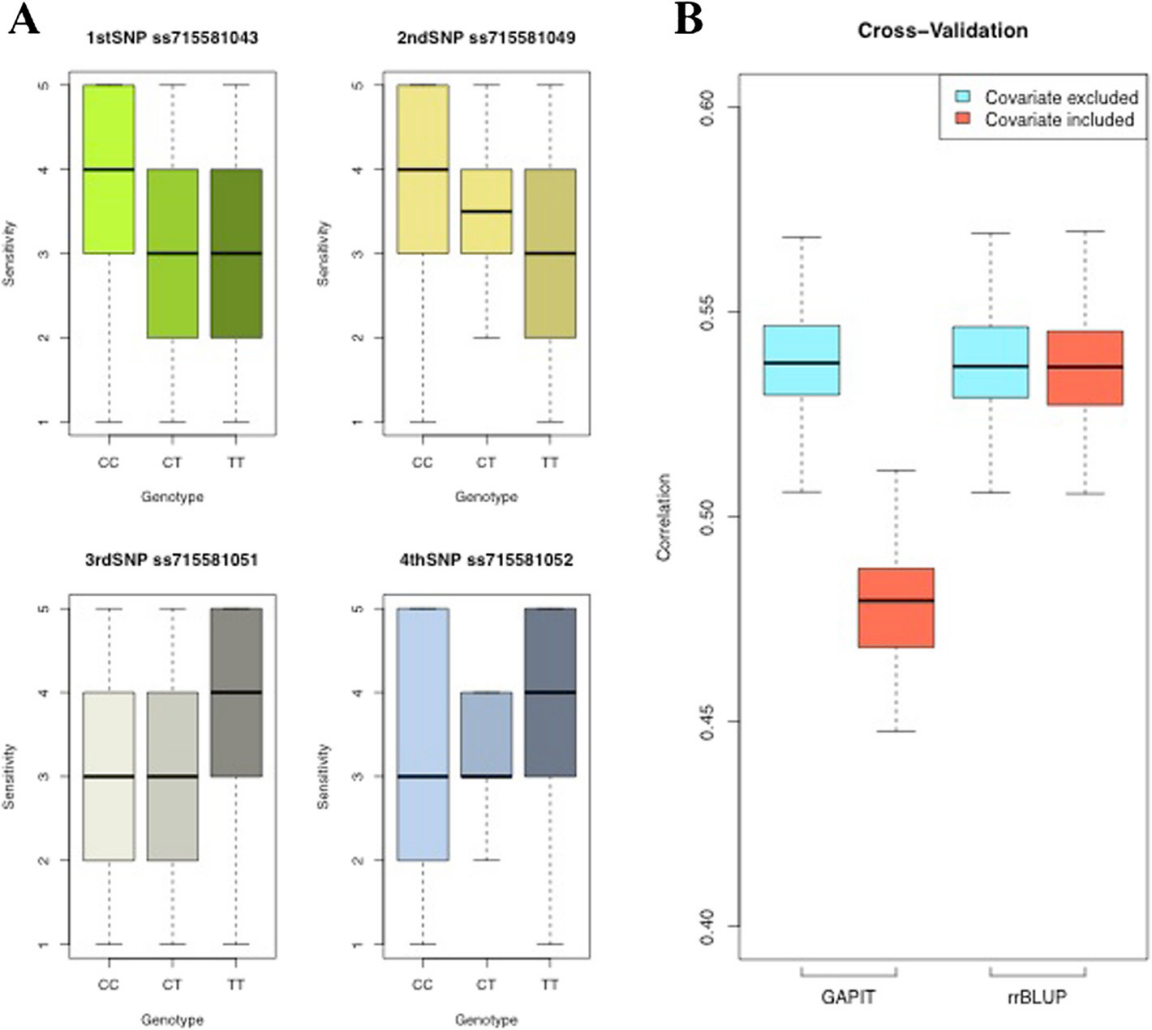
Mean separation by significant SNPs and cross-validation by GAPIT and rrBLUP. **a** Distribution of TRSV sensitivity by each significant SNP genotype showed strong overlap between the sensitivity scales. **b** Five-fold cross-validation with or without the most significant SNP, ss715581043, as the covariate. Cross-validation was evaluated by GAPIT and rrBLUP. In general, the correlation of training and validating population dropped slightly when the covariate was included in the model for both GAPIT and rrBLUP

**Fig. 5:**
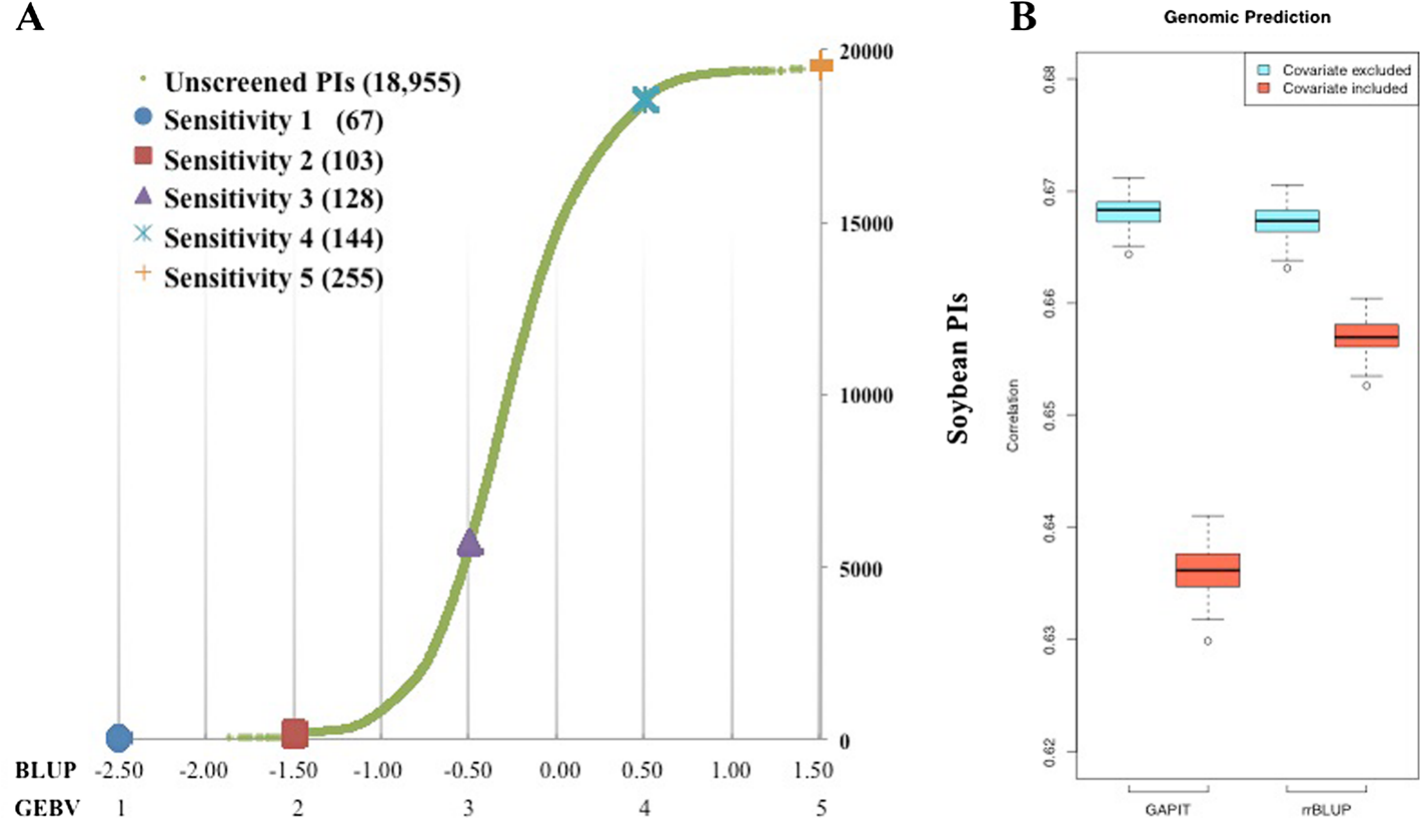
Genomic prediction and accuracy evaluation.**a** BLUP and GEBV for 18,955 unscreened soybean PIs by GAPIT. GAPIT generated a continuous BLUP value for 18,955 soybean PIs, while BLUP for the training population (with determined TRSV sensitivity) were centered at−2.50 (GEBV 1),−1.50 (GEBV 2),−0.50 (GEBV 3), 0.50 (GEBV 4) and 1.50 (GEBV 5). **b** Comparison of the TRSV sensitivities of the selected 55 soybean accessions that were selected from the predicted 18,955 soybean accessions resulted in correlation between 0.63 and 0.67. The evaluations by GAPIT and rrBLUP had similar results and the inclusion of the most significant SNP, ss715581043, as the covariate reduced the correlation slightly

Because a single genomic region on chromosome 2 associated with TRSV sensitivity, the mean separation of TRSV sensitivity at each of the four most significantly associated SNPs were tested to determine if marker-assisted selection would aid in breeding for reduced sensitivity to TRSV infection. The results suggested strong overlaps in these distributions (Fig. 4a), and the correlation between genotypes of each SNP to the rated TRSV sensitivity resulted in low significant (*P* > 0.01) correlations (*r* = 0.15, *r* = 0.14, *r* = −0.14, *r* = −0.15 for the first, second, third, and fourth SNPs, respectively). We then tested if genomic selection would provide a better prediction and applied GAPIT and rrBLUP to perform cross-validation [23, 24]. The results of the five-fold cross-validation without a covariate in the model resulted in significant correlations (*r* = 0.54) for both GAPIT and rrBLUP. When the most significant SNP, ss715581043, was fixed as a covariate in the model, the correlation coefficient was reduced for GAPIT (*r* = 0.48) but not rrBLUP (*r* = 0.54) (Fig. 4b). These results indicated that genomic prediction without including a covariate might provide a better estimate for the unscreened soybean accessions in the USDA Soybean Germplasm Collection. We subsequently conducted genomic prediction by splitting the unscreened 18,955 soybean PIs into 85 groups to increase computational speed and acquired an average BLUP for each of the 18,955 soybean PIs. The BLUPs of the unscreened soybean PIs was sigmoidal with continuous values while the BLUP of the training subpopulations were centered around − 2.5,−1.5,−0.5, 0.5, and 1.5 that corresponded to genomic estimated breeding values (GEBVs) of one to five, respectively (Fig. 5a). The distribution of BLUPs and GEBVs for the 18,955 unscreened soybean PIs suggested none was close to PIs in the TRSV sensitivity one from the training population (Fig. 5a; Additional file 1: Table S1). We then phenotyped an additional 55 PIs selected from the predicted GEBV distribution for sensitivity to TRSV in order to evaluate the accuracy of genomic prediction (Fig. 5a). The GEBV showed that the TRSV sensitivities of the 55 PIs: 19 PIs with TRSV sensitivities of two (1.5 ≤ GEBV < 2.5), 15 PIs with TRSV sensitivities of three (2.5 ≤ GEBV < 3.5), 13 PIs with TRSV sensitivities of four (3.5 ≤ GEBV < 4.5), and 8 PIs with TRSV sensitivities of five (GEBV ≥ 4.5). The rated TRSV sensitivity was correlated (*r* = 0.67, *P* <  0.001) to GEBVs obtained from both GAPIT and rrBLUP (Fig. 5b). However, the accuracy of genomic prediction tended to be divergent in the lower ratings but more reliable toward the higher sensitivity ratings. That is, most plants with predicted sensitivity ratings of five and four had actual sensitivity ratings of five and four, but accessions with a predicted sensitivity rating of two had actual sensitivity ratings that ranged from one to four (Table 4).

**Table 4:** GEBV and actual TRSV sensitivities of 55 soybean plant introductions

Plant introduction	GEBV^a^	Actual Sensitivity	Plant introduction	GEBV^a^	Actual Sensitivity
PI594142	1.72	3	PI092470	3.08	5
PI548527	1.75	4	PI511361	3.09	4
PI634903	1.82	3	PI603327	3.13	3
PI548598	1.87	4	PI468400B	3.38	5
PI603290	1.95	3	PI416864	3.40	5
PI548525	1.97	3	PI548301	3.42	4
PI578479	1.99	4	PI399066	3.46	3
PI602449	1.99	4	PI192871	3.47	5
PI088289	2.00	3	PI417536B	3.49	4
PI088290	2.00	3	PI594418B	4.04	5
PI592933	2.04	3	PI603633	4.09	5
PI594399C	2.06	2	PI594570B	4.12	4
PI092713	2.07	4	PI437207	4.14	5
PI634890	2.16	1	PI587880B	4.16	5
PI518677	2.36	3	PI594837A	4.19	4
PI591503	2.37	3	PI436566	4.21	4
PI547878	2.40	4	PI587554	4.28	5
PI547663	2.41	2	PI594635B	4.29	5
PI091733_1	2.43	4	PI341254	4.44	5
PI507709	2.45	3	PI103080	4.63	5
PI547821	2.48	4	PI603449	4.69	5
PI547531	2.51	4	PI603524	4.69	5
PI060269_2	2.54	2	PI407739	4.81	5
PI408335A	2.96	2	PI253664	4.85	5
PI438501	2.98	2	PI603459	4.85	5
PI399004	3.00	2	PI603451A	4.87	5
PI437607	3.02	4	PI603581	4.89	5
PI592974	3.07	3			

^a^GEBV was an average of 10 predictions performed by GAPIT

## Discussion

In this study, 697 of the 19,652 soybean PIs in the USDA Soybean Germplasm Collection were evaluated for their responses to TRSV inoculation. Although no TRSV-immune accessions were observed, 43 % of the accessions did not display bud blight, the most severe symptom. A GWAS identified a single genomic region on chromosome 2 strongly associated with TRSV sensitivity using the unified mixed linear model [25] in the GAPIT package [24], which identified a 130 kb chromosome 2 interval that contains Glyma02g13460 and Glyma02g13470, two candidate LRR-RLK genes.

Genes of the LRR-RLK type are well known for their involvement in pathogen-associated molecular pattern (PAMP)-triggered immunity (PTI). Classical examples are the ligand-dependent receptor Flagellin Sensing2 (FLS2) that recognizes bacterial flagellin and EF-TU Receptor (EFR) that recognizes EF-TU. After recognizing the extracellular PAMP, another RLK co-receptor (BRI1-Associated receptor Kinase1/Somatic Embryogenesis Receptor-like Kinase3) forms a complex with FLS2 or EFR and activates downstream PTI defense responses [26]. One of the LRR-RLK genes from *A. thaliana*, *ERECTA*, is particularly interesting for its involvement in developmental processes in multiple tissue types, including aerial organs, epidermal tissue, pedicels, and floral primordia, as well as its function in controlling resistance against the bacterial pathogen *Ralstonia solanacearum* (Smith) Yabuuchi [27], fungal pathogen *Plectosphaerella cucumerina* (Lindf.) Arx and oomycete pathogen *Pythium irregular* Buisman [28]. It has been proposed that *ERECTA*-dependent resistance against necrotrophic *P. cucumerina* is linked to regulation plant cell wall biosynthesis [29].

In addition to ERECTA that is associated with bacterial and fungal resistance, nuclear shuttle proteins (NSPs) from viruses in the *Geminiviridae* have been reported to interact with *A. thaliana* NSP-interacting kinase1 (NIK1), which also belongs to the LRR-RLK protein family [30]. Unlike TRSV, viruses in the *Geminiviridae* have bipartite circular single-stranded DNA genomes (DNA-A and DNA-B). While DNA-A of *Geminiviridae* encode proteins involved in replication, transcription, and encapsidation, DNA-B encodes two proteins, NSP and a movement protein. The NSP inhibits the kinase activity of NIK1 by binding to the kinase active site and activation loop, which contains an essential threonine residue (T474). NIK1 deletion mutants displayed enhanced susceptibility to viruses in the *Geminiviridae* [31], and ectopic expression of a nonphosphorylatable NIK1 in the NIK1 deletion mutant failed to rescue the enhanced virus susceptibility [32]. Blocking of T474 eliminated its kinase activity and abolished the phosphorylation of a protein that moves from cytosol to the nucleus when phosphorylated, where it interacts with a nucleus-located transcription factor to down regulate translation processes that eventually suppress viral replication [33]. Under the proposed mechanism, NIK1 serves as a target to NSP for suppressing host defense responses. The discoveries of how NIK1 is involved in controlling plant susceptibility to geminiviruses may underline how one or two soybean LRR-RLK genes in the chromosome 2 region harboring peak genomic associations with TRSV sensitivity could control the disease responses to TRSV. If these two LRR-RLK genes are confirmed to play a biological role in regulating TRSV sensitivity, it may imply that these soybean LRR-RLK are virulence targets for TRSV that control sensitivity rather than resistance, if the mechanism of the interactions of soybean LRR-RLK proteins with TRSV are similar to NIK1 with geminiviruses. Further studies focused on comparing the protein sequences of the two LRR-RLK genes from soybean PIs with different sensitivity levels to TRSV may reveal if any amino acid polymorphism indeed associate with these levels of sensitivity. In addition, characterization of the putative viral component(s) and the mechanism of interaction may improve our understanding on how soybean sensitivity to TRSV is controlled.

Genomic prediction has become a powerful tool for rapidly predicting plant phenotypes based on genome-wide marker information. This approach has great potential to accelerate plant breeding cycles because it requires fewer generations of selection compared to phenotype-based breeding approaches [34]. A recent review on “next generation breeding” illustrated how next generation sequencing will be used to more quickly improve crop productivity [35]. Multiple genomic prediction models have been developed with similar accuracies [36], and in our study, we applied the compressed BLUP approach in the GAPIT R package [24] and the ridge regression BLUP in the rrBLUP R package [23]. Although a single locus on chromosome 2 was identified for TRSV sensitivity, our results suggest that genomic prediction performs better than marker-assisted selection. We showed that the prediction accuracy among the additional 55 soybean PIs was close to that obtained from the cross-validation study of the 697 PIs used for the GWAS. Moreover, we noticed the prediction was more accurate and conservative in identifying soybean accessions that displayed severe necrosis symptoms. There are several limitations of genomic prediction, and one of them is the possibility that the phenotype is a combination of genetic and environmental effects [37]. It has been reported that soybean response to TRSV may differ by maturity stage [38]. Accordingly, it is possible that there may be more soybean PIs in the USDA Soybean Germplasm Collection that have TRSV sensitivity of one but may have been misjudged by the genomic prediction.

## Conclusion

TRSV is a potential threat to the soybean industry with limited resistance to the virus identified. To understand if additional resistance exists in the USDA Soybean Germplasm Collection, we evaluated 697 soybean accessions for sensitivity to TRSV infection. By performing a GWAS using the publicly available SoySNP50K marker set, we identified a novel genomic region on chromosome 2 containing two candidate LRR-RLK genes that may control sensitivity to TRSV. We also assessed the ability of the SoySNP50K markers to predict TRSV sensitivity for 18,955 soybean PIs in the USDA Soybean Germplasm Collection, and high prediction accuracies were obtained. Our study not only discovered a new locus for TRSV sensitivity but also demonstrated the potential of using GWAS and genomic prediction for genetic analysis with the use of the SoySNP50K resource.

## Methods

### Phenotyping and genotyping soybean PIs

Soybean accessions used in this study were obtained from the USDA Soybean Germplasm Collection (http://www.ars-grin.gov/npgs/). Soybean plants were grown in a growth chamber at 25 °C and inoculated with TRSV using carborundum as an abrasive at the unifoliate stage around 10 days after sowing. Inoculated plants were kept in a moist chamber at 25 °C for about 16 h, and returned to a growth chamber set at 25 °C. Sensitivities were scored based on a sensitivity scale from one to five at 10 days after inoculation with one showing the least amount of symptoms and five showing the strongest symptoms including bud blight (Fig. 1a). A completely randomized design was used to test sensitivity of PIs with a minimum of three plants per trial. There were three trials completed over time using a different randomization for each trial. SoySNP50K was downloaded from SoyBase (http://www.soybase.org), and split into 20 profiles based on chromosomes. For each profile (chromosome), missing SNPs were imputed by BEAGLE version 3.3.2 [39]. There were overall 42,449 SNPs available but SNPs with minor allele frequencies below 0.1 were excluded, leaving 30,697 SNPs for GWAS.

### GWAS and genomic prediction

Five PCs were used in a BIC-based model selection procedure that determined how many PCs were needed to control for population structure in the unified mixed linear model used for the GWAS. A kinship matrix was calculated by the VanRaden method using mean and average cluster algorithm [24]. GWAS was conducted in GAPIT using the unified mixed linear model including the kinship matrix but excluding PCs [25]. A total of 697 soybean PIs rated for TRSV symptom sensitivity were included in the GWAS (Additional file 2: Table S2). Given the inherent conservativeness of correcting for multiple testing in a GWAS, two multiple testing procedures were implemented. The Benjamini-Hochberg (1995) procedure was used to control the FDR at 1 %, and the Bonferroni procedure was implemented to control the experiment-wise type I error rate at 0.05. To search for any possible minor signals, the most significant SNP (ss715581043) was fixed as a covariate.

To determine if marker-assisted selection could predict TRSV sensitivity, numeric genotypes of each significant SNP among the 697 soybean PIs were correlated to their sensitivity. To determine if genomic selection could predict TRSV sensitivity, five-fold cross validation, with or without a covariate in the model, was tested using GAPIT and rrBLUP. In each five-fold cross-validation, 140 soybean PIs were assigned to a validation population, and the remaining 557 soybean PIs were used as training population to build the model. Each accession of the 697 soybean PIs was assigned once as the validation population in a five-fold cross-validation. The mean of each five-fold cross-validation, which is a correlation between the BLUPs of validation population that generated from the training model and the TRSV sensitivity of validation population, was saved as a result of a five-fold cross-validation. A total of 100 iterations of five-fold cross-validation were conducted with the 697 soybean PIs randomized in order for each run. The same five-folds were used to assess the predictive accuracy of the genomic prediction models used in GAPIT and rrBLUP. The mean of the 100 iterations was presented to represent the results of GAPIT and rrBLUP.

To assess the predictive accuracy of genomic prediction in the remaining 18,955 unscreened soybean PIs in the USDA Soybean Germplasm Collection (Additional file 1: Table S1), these PIs were randomly divided into 85 groups with 223 PIs per group to reduce computational time. To predict GEBVs for the unscreened groups, the genotypes of each group were combined to the genotypes of the 697 screened PIs to fit a genomic prediction model in GAPIT; thus 85 independent genomic prediction models were fitted to acquire one GEBV for each of the 18,955 PIs. A total of ten runs for each of the 18,955 unscreened soybean PIs were conducted and the mean was used to represent the GEBV for each of the PIs. To approximate the genomic prediction accuracy, a total of 55 soybean PIs were selected from the 18,955 unscreened soybean accessions and phenotyped for their actual TRSV sensitivities following the methods described above. Cross-validation was conducted as described above to obtain a mean correlation between predicted GEBV and actual sensitivities of these 55 accessions, which was regarded as the prediction accuracy for genomic prediction.

### Availability of data and materials

The original genotypic data (SNPs) used in this study are available on SoyBase (http://www.soybase.org); the original phenotypic data for association mapping and genomic prediction are available in supplementary Table 1; and the analyzing tools, GAPIT and rrBLUP, are available on developer’s website (http://www.maizegenetics.net/#!gapit/cmkv) and R CRAN website (https://cran.r-project.org/web/packages/rrBLUP/), respectively.

## Additional files

Additional file 1: Table S1. LUP and GEBV of unscreened 18,955 USDA Soybean Germplasm Collection. (XLSX 890 kb) Additional file 2: Table S2. T RSV sensitivities of 697 soybean PIs that used for GWAS. (XLSX 45 kb)

## Abbreviations

BIC: Bayesian information criterion
BLUP: Best linear unbiased predictor
FDR: False discovery rate
GAPIT: Genome association and prediction integrated tool
GWA: Genome-wide association studies
LRR-RLK: Leucine-rich repeat receptor-like kinases
PC: Principal components
PI: Plant introduction
QQ: Quantile-quantile
QTL: Quantitative trait loci
SNP: Single nucleotide polymorphism
TIR-NB-LRR: Toll/interleukin-1 receptor, nucleotide-binding site and leucine-rich repeat
TRSV: *Tobacco ringspot virus*
